# Deviation from Nash mixed equilibrium in repeated rock–scissors–paper reflect individual traits

**DOI:** 10.1038/s41598-025-95444-6

**Published:** 2025-04-29

**Authors:** Kensuke Arai, Suma Jacob, Alik S. Widge, Ali Yousefi

**Affiliations:** 1 Computer Science Department, Worcester Polytechnic Institute, Worcester, MA 01609 USA; 2 Semel Institute, Child & Adolescent Psychiatry, University of California, Los Angeles, CA 90210 USA; 3 Department of Psychiatry and Behavioral Sciences, University of Minnesota, Minneapolis, MN 55414 USA; 4 Department of Biomedical Engineering, University of Houston, Houston, TX 77204 USA

**Keywords:** Cognitive neuroscience, Social neuroscience, Human behaviour

## Abstract

Current psychiatric nosology is based on observed and self-reported symptoms. Heterogenous pathophysiological mechanisms may underlie similar symptoms leading to diagnosis not matching up to the neurobiology. Recent research has sought to move away from diagnoses by symptoms, to viewing aberrant mental health in terms of abnormal human neurobehavioral functioning and concurrent deviations in the pathophysiology. Human behavior in a social context is a core neurobehavioral function with large individual variation that may reflect genomic, metabolic or neurobiological variation, whose identification potentially yields more accurate targeting for the development of interventions and biomedical treatments. In this research, we describe an experimental framework that utilizes a zero-sum game of repeated Rock–Paper–Scissors played against an artificial intelligence agent as an assay of social interaction. Human deviation from the Nash Mixed Equilibrium strategy of play, the only guaranteed way to avoid exploitation, can be seen in the sequential dependence of hands. We hypothesize that this deviation represents humans mimicing randomness to avoid exploitation through constant adjustments of behavior, which we analyze in terms of a set of switching heuristic lag-1 conditional response rules. We quantify and interpret the set of rules subjects are able to utilize as mirroring individual traits. Subjects in the study also completed the Autism Quotient Abridged survey, and subscores of the social, imagination and routine factors were found to be predicted by a combination of behavioral features derived from game play.

## Introduction

Symptoms of psychiatric illness often manifest simultaneously and on a continuum at many physiological, neurological and cognitive levels. Current clinical nosology is based on a profile of symptoms generated from self-report and assessment by family members, teachers or psychiatrists^1–4^, but biased response in self-report^5^, low inter-rater reliability^6–8^, social desirability bias^9^ and inherent subjectivity, are known drawbacks to these assessments, demonstrated by Rosenhahn’s sensational experiment^10^. Further, heterogenous pathophysiological mechanisms may underlie similar symptoms, leading to classification of disorders that do not match up to the causal neurobiology, one possible reason leading to low response rate of psychiatric drugs^11^. The National Institute of Mental Health has introduced the Research Domain Criteria (RDoC), a framework of new approaches to mental health research that views mental health and psychopathology in the context of major domains of basic human neurobehavioral functioning, and considers psychopathology in terms of deviations from normal functions that could account for particular symptoms. Researchers are developing new assays that measure the functional outputs of a patient’s neurobiology like auditory responses^12,13^, motion perception^14,15^, cross-modal audio-visual perception^16^, spatial temporal perception^17^, visual social cue perception^18,19^, behaviors observation of repetitive behavior^20^ and body posture^21^. It is anticipated that identification of patients with functional outputs that lie far from the norm, coupled with genomic and metabolic biomarkers^22–24^, and resting state brain dynamics^25–28^, will result in diagnostic categories with included patients with a more homogenous psychopathological mechanism.

Impairment of social function is a common symptom of psychiatric illness. The full extent of deficient social functioning may only manifest itself in actual complex, reciprocal social interactions^29^, which are difficult to reproduce repeatably in a clinical setting as minimally, a human dyad is required. It is unrealistic to pair every test subject against the same partner, and while interaction with a computer can make a task repeatable, reciprocal interactive laboratory tasks are still rare^30^. Neuroecomonic games^31^ have been used to investigate decision making tasks in a social setting, but present unique challenges not present in the analysis of isolated or programmatically illicited behaviors. To address these concerns, we propose using an AI-backed interactive competitive neuroeconomic game^32^, that allows study of social interaction, and a relevant and succinct analysis and modeling framework that captures a subtle but robust mechanism of adaptive social behavior. Games restrict the degree of freedom of environment and actions that allow easy state description, yet may still exercise some of the same social cognitive skills, the “theory of mind” required in natural social interaction like reading anothers intentions^33,34^. We argue replacing human opponents with an AI agent is a good surrogate to pairing the same test opponent to every participant, permitting repeatable social experiments with reciprocal interaction across a participant population to be studied. Further, a game would also allow concurrent monitoring of time-locked biological signals that may elucidate how both behavior and cognitive process differ among affected individuals.

**Fig. 1:**
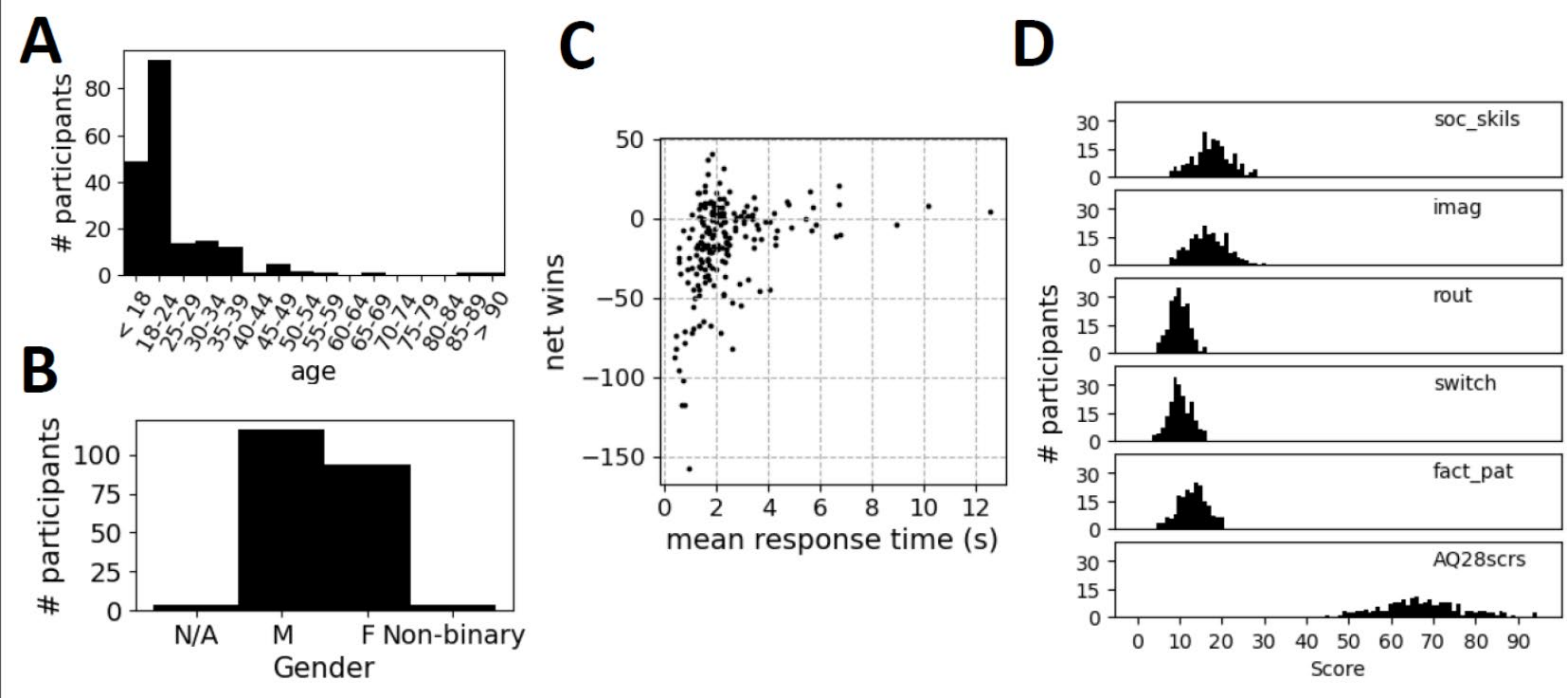
Demographic and basic game statistics of HPvAI(AQ) participants. (**A**) Age and (**B**) gender distribution of participants. (**C**) Net wins versus mean response time (round duration). (**D**) Distribution of Abridged Autism Quotient subscores and composite score. Bottom figure is the sum of top 5 subscores.

**Fig. 2:**
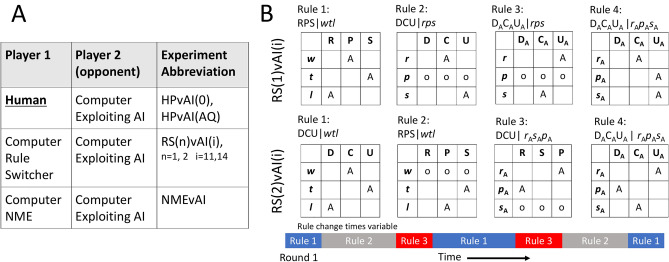
Experiment information. (**A**) List of abbreviations used for experiments and simulations, and the identities of player 1 and 2. Data from human players HPvAI(AQ) $\subset$ HPvAI(0). (**B**) Rules used in RS(1)vAI(i) and RS(2)vAI(i) simulations. $i=11$ or 14 indicate the mean interval in rounds between rule changes. The conditional probability matrices for each ruleset shown, with A=0.9, empty square=0.05, and o=1/3. Rules where one action nearly deterministic represents repertoire rule, for example $p(\text{ C }|r)=A$ in Rule 2 of RS(1)vAI(i), while there are a few conditions where all the actions are set to be equiprobable, like the $p(\text{ D,C,U }|p)=o$ in Rule 2 of RS(1)vAI(i) to simulate a possible case where player showed little serial dependence for that condition. A cartoon of these rules being switched in simulation for RS(n)vAI(i) shown below probability matrices.

Rock–Paper–Scissors (RPS) is a popular game worldwide often used to settle playground disputes. After one round, the loser might move the goalpost and declare “best of 5”. In longer games of repeated Rock–Paper–Scissors (rRPS), any pattern or bias a player exhibits becomes open to exploitation. The Nash Mixed Equilibrium (NME) in rRPS chooses R, P or S at random with equal probability, and is the only guaranteed strategy that avoids exploitation. Humans are poor generators of random behavior^35,36^, and investigation has focused into how human behavior deviates from the normative standard of the NME. Evidence of deviation initially focused on whether marginal action distribution is biased away from equiprobability, with some investigators finding a slight bias towards R^37–39^, although the results were not consistent across studies^40^. Another potential point of deviation is in the presence of sequential dependence in the moves, and studies have typically looked for one of several commonly considered dependency rules. The most widely studied ruleset is the operant conditioning principle of win-stay-lose-switch, an approximate algorithm for Bayesian reinforcement learning^41–43^. Forder et al.^42^ found that the degree of fidelity to win-stay probability is flexible as the reward value is adjusted in non-zero sum versions of rRPS, while the lose-switch probability remains unchanged. Srihaput et al.^43^ found that when playing repeated rounds against a randomly assigned opponent, win-stay probability drops but lose-switch probability remains unchanged when comparing consecutive rounds where the opponent remained the same versus consecutive rounds where opponent was changed. These results suggest different neural pathways that generate the actions following win and lose, which may be an evolutionary behavioristic consequence of the higher risk to the organism of repeating failures than experimenting with conditions leading to success^42,43^. In an experiment where several 6-player groups played rRPS, players were randomly re-assigned a partner from within their group. A slight, group-level bias for one hand that slowly cycled in order (R→P→S→R→P→S or reverse) emerged, and the cycling period of each group could be explained by the mean win-stay-lose-switch probabilities of that group^38^, demonstrating an observable consequence of sequential dependency in player moves. Other heuristic rules like cycling and Cournot best response (choose hand that beats last opponent hand) have been considerred, and schizophrenia patients have shown pronounced preference for ascending cycling against computer opponent, and choosing the Cournot best response against human opponent^37^. Most of these studies find the population mean of these probabilities to be near 1/3, which is not a strong indication of serial dependence, yet serially dependent heuristic strategies have been codified by expert players (WRPSA website:“Strategies”) like “win-stay, lose switch”, mimicry (ie copy opponent’s last move or beat opponent’s last move) and cycling. Zhang et al.^44^ has found subpopulation among study participants of those who play win-stay-lose-switch, and those who play win-switch-lose-stay, suggesting not only the importance of considering the individuality of subjects, but also the possibility of traits being reflected in the indiviuality of game play.

**Fig. 3:**
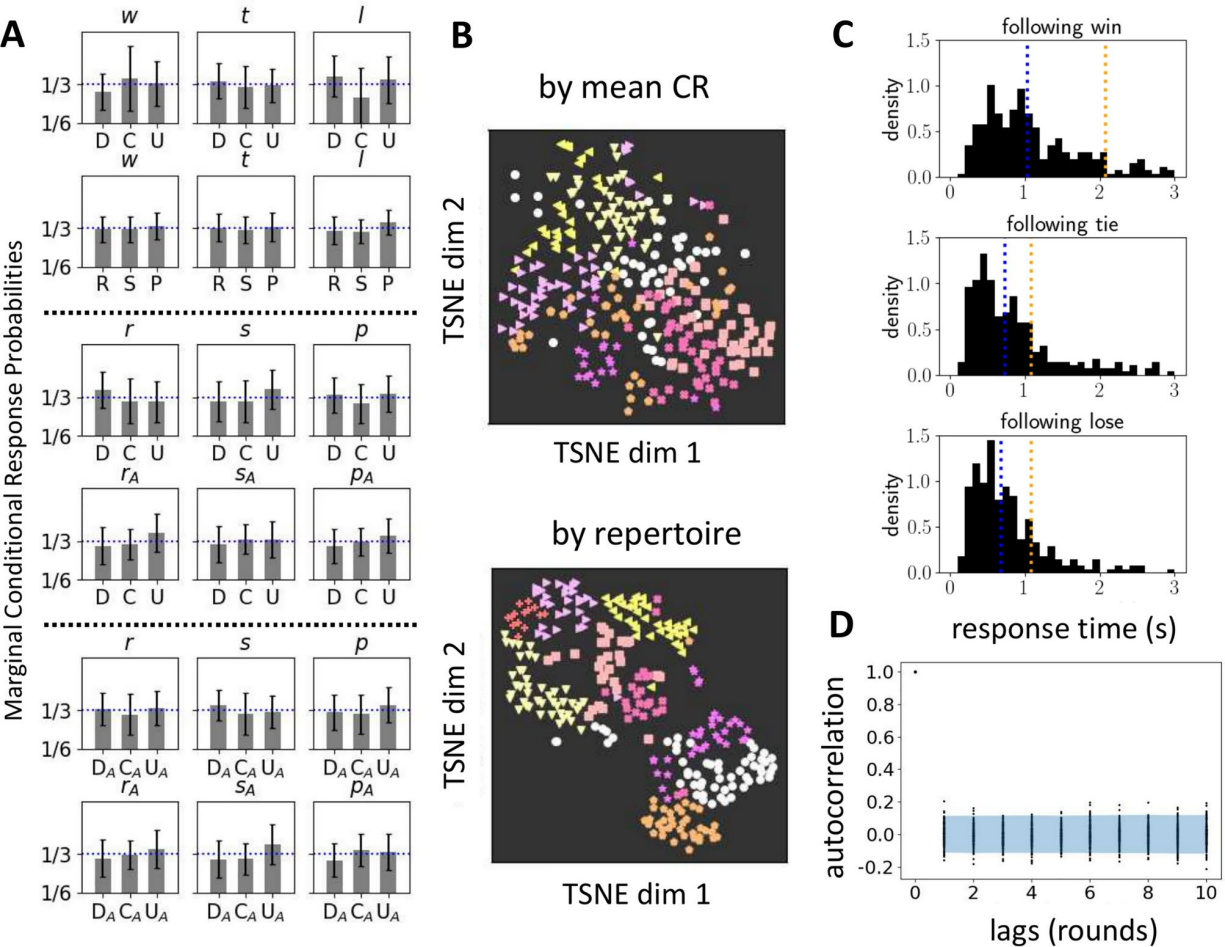
Reproduction of key existing results using HPvAI(0) population. (**A**) Population mean conditional response probabilities calculated over the entire game for each of the 6 frameworks. Conditions indicated in lower-case italic above, actions in upper case below. Most components are near 1/3. (**B**) Low-dimensional embedding of the the conditional responses using t-SNE, color-coded by cluster labels obtained by agglomerative clustering, top. The embedded data do not appear clustered in comparison with the embedded RSSs used in detection of in-repertoire rules, reproduced below from Fig. 4C, where clusters in the embedded data are visible. (**D**) Response times after wins, ties and losses. Blue and orange are mean and median, respectively. (**E**) Auto-correlation of the win timeseries. Each point represents lagged auto-correlation for a single HP, and the blue band is the 95% confidence region for null distribution obtained by shuffling round orders.

A young child might play a string of Rs, and our ability to recognize this and counteract it, is an example of reasoning about the competitor and exploiting a glaring regularity. On the other end of the spectrum, macaques playing against computers programmed to play NME end up playing the same hand over 90% of the time^45^, a realization perhaps that there is no regularity in the opponent, and also that the opponent will not exploit even the most obvious regularity. Batzilis et al.^46^ has found that in first-time encounters with an opponent, players are closer to the NME, but upon subsequent encounters, use accrued information about them to deviate from the NME, and are able to exploit opponents. Brockbank et al.^47^ has investigated classes of regularities humans can exploit by pairing humans against computer opponents that were programmed to exhibit sequential regularity. Human players displayed a wide ranging difference in how successfully different rules were exploited, but typically advantages were not visible until tens of rounds were played. Stöttinger et al.^48^ has found that adapting to a dynamic competitor occurs more efficiently when the competitor changes to structurally similar rules. These results suggest that human players adapt, and are not strictly following any one heuristic, as doing so would open the player up to exploitation. Inference of regularities and adaptition to sudden environmental changes are also present in tasks like Wisconsin Card Sorting Test^49,50^, but rRPS differs in the reciprocity present in the competitive interaction. Most studies have limited investigation of sequential dependence to the most recent past round, and have not addressed this adaptation. Brockbank et al.^40^ has found no evidence of win streaks or losses that are expected if adaptation occurred transiently and on a short timescale^51^, leading to the hypothesis that humans adapt over long periods to aggregate patterns of play, and considered dependencies that can potentially reach further into the past beyond the last round.

We hypothesize that rather than a slow adaptation process, heuristic used in game play changes abruptly on a scale of a few rounds^51^, possibly to mimic randomness. We justify this view based on our observation that slow adaptation is likely not sufficient to outpace the fast, constant learning of our AI. To support this alternative view, we identified what heuristic was being used at all times during the game, and detected the timing of when heuristics change, and found changes coincide with a sharp regaining of advantage against the AI, whose most recent learned behavior of the human player suddenly becomes obsolete following an abrupt change, effectively mimicing randomness. In the context of rRPS, we found that the repertoire of rules which a player chooses from is characteristic of each player, and details about the repertoire are predictive of ASD traits as measured by the Abridged Autism Quotient answered by each subject. This is reminiscent of reports of individuality in human-generated random sequences^35,36^ and its predictive link to capacity for creative thinking^52^. Autism is a potential use case of the rRPS as a social behavioral assay, as deficits in social function is a defining feature of autism, though this behavioral assay may also be sensitive to deficient social function in other disorders. Because rRPS is a purely competitive game, it only explores competition unlike mixed-motive games like Stag Hunt^34^ or Prisonner’s Dilemma^53^ where cooperation is also a viable strategy as in many real-world social scenarios. Nevertheless, the rich space of heuristic rules that can be used in rRPS suggests the game is still a promising assay of individual social behavioral traits.

## Methods

### Experiment

1370 anonymous volunteers (human players, HP) were recruited from the online cognitive experimental platform testmybrain.org^54^ between July to December 2021 to play 300-round games of rRPS (cyclic dominance rules rock beats scissors, paper beats rock, scissors beats paper) against an AI agent running on a web browser, playable on iOS, Android, Windows and Macintosh. The study was approved by the Institutional Review Board of Worcester Polytechnic Institute, Protocol Number IRB-21-0568. The authors confirm that this experiment was performed in accordance with the approved guidelines and regulations, and informed consent was obtained from all the human participants. The AI was comprised of 3-perceptrons each representing R, P and S^55,56^, whose internal parameters were updated online after each round, and that predicted the next HP move using the past 2 HP and AI moves. The rounds were self-paced, and a noticeable degradation of performance was observed for participants who spent less than 600ms per round, Fig. 1C, close to human reaction time of around 250ms, leading us to suspect lack of engagement in such participants, and they were subsequently removed from analysis. More than 15 consecutive repeated responses from the HP was also deemed as lack of engagement. In addition, because the response of the AI is strongly history-dependent, as is heuristic rules used by HP, participants were also removed if there were more than 10 round-round intervals > 1 minute. In addition to playing the 300-round game, participants also completed the 28-item Abridged Autism Quotient^4^ (AQ28), whose composite score is a sum of 4 subscores assessing personality traits characteristic of ASD - Social Skills (8 items), Imagination (8 items), Routine (3 items), Switch (4 items) and a subscore assessing fascination with Numbers and Patterns (5 items), Fig. 1D. The AQ28 is a self-administered questionnaire that does not address symptoms of mental or neurodevelopmental disorder, but that identifies and assesses personality traits correlated with the ASD spectrum that shows continuous distribution in the healthy population.

This left 298 players who completed a 300-round game and were deemed credibly engaged most of the time, and a 193-player subset who also responded to the AQ28 survey. We refer to these populations as HPvAI(0) and HPvAI(AQ), Fig. 2A, respectively. Most of the participants were in their early 20s, with slightly more identifying as males than females, Fig. 1A,B. The mean net wins over a round for these HPs was -15.4, with standard deviation 28.1, Fig. 1C. The participant mean AQ28 of our participant population was 67.7, which compares to a mean of about 55 for healthy populations and 85 for autistic populations reported by Hoekstra et al.^4^.

**Fig. 4:**
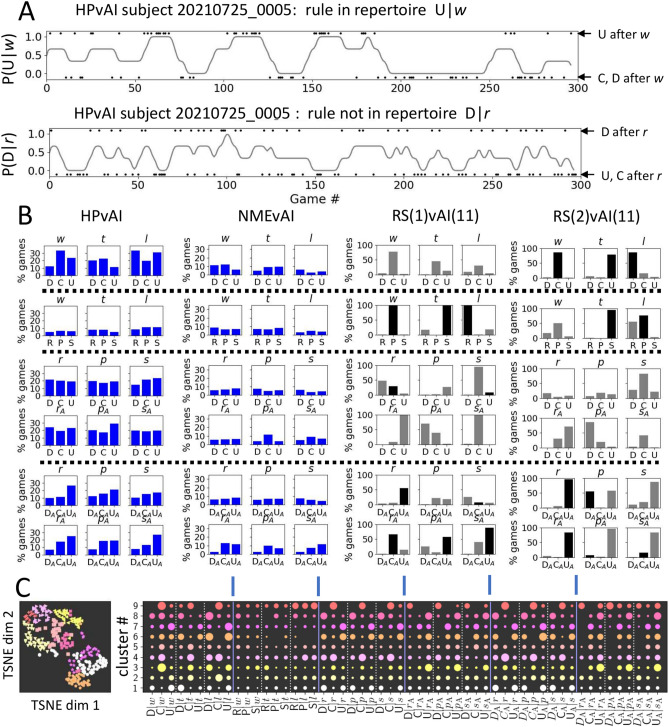
Detection of rule repertoire. (**A**) Example traces of the dynamic conditional response probability calculated for 2 of the possible 54 rules from a HPvAI(28) participant, with top rule classified as in-repertoire. Conditioning events (black dots) plotted at top and bottom represent occurrences that match or do not match the rule, respectively. Rules in-repertoire tend to have interspersed, extended periods where probability is near 1, while rules not in-repertoire rarely go near 1. (**B**) Fraction of the population in which each of the $6\times 9$ rules were classified as in-repertoire for experiments HPvAI(0), RS(n)vAI(11) and NMEvAI. Ground truth knowledge of which rules are in the repertoire for RS(n)vAI(11), plotted as a black bar. For the NMEvAI, near chance level detection equally across all rules, as expected. The dotted lines organizes the figures by their transitions to assist in visualizing the pattern present in HPvAI. (**C**) There are stereotypical patterns in the heterogeneity of the repertoire in HPvAI(0). The 54 RSSs used in detection of in-repertoire rules for all the subjects were projected onto a 2-dimensional manifold using t-SNE. Spatial clusters with significant empty space between them in the 2-D distribution of points well matches the colored labels assigned by Agglomerative Clustering set to find 9 clusters, left scatter plot. The pattern of rules that are in-repertoire for each cluster shown on the right. Circle size indicates fraction of subjects in the cluster where rule was in-repertoire.

### Lag-1 rules

Lag-1 conditional response rules are rules of the form “take action Y if condition X occurred in previous round”. In the context of rRPS, we consider 3 natural sets of triggering conditions that occur within the game: 1) HP’s own hand (***r***ock, ***p***aper, ***s***cissors), or 2) AI’s hands ***r***_A_ock, ***p***_A_aper, ***s***_A_cissorrs, or 3) the outcome of HP and AI’s hands ***w***in, ***t***ie, ***l***ose. We do not consider finer-grained conditions like “won (tied) (lost) using rock (scissor) (paper)”. HP’s actions that follow can be 1) the next hand itself **R**ock, **P**aper, **S**cissors, or transition with respect to the 2) player’s or 3) AI’s last hand **D**owngrade or **D**_A_owngrade, ie choose move that loses to own or AI’s last hand, **C**opy or **C**_A_opy, ie repeat own or AI’s last hand, or **U**pgrade or **U**_A_pgrade, choose move that beats own or AI’s last hand. By combining a conditions and actions set, a ruleset can be created that generates the next action. For example, combining outcome and transitions from HP’s last hand can result in a deterministic ruleset “**U**pgrade after ***w***in”, “**C**opy after ***t***ie” and “**D**owngrade after ***l***ose”. Or combining AI’s last hand as conditions and directly specify a hand as an action result in a set of rules like “**P**aper after ***r***_A_”, “**P**aper after ***p***_A_” and “**R**ock after ***s***_A_”. These sets of rules picks a move that beats the AI’s last move, except when AI plays “***p***_A_”, in which case it copies the AI. Because there are 3 actions that can be taken in rRPS, the above rule statements are actually 3 probabilistic rules *p*(R$$|r)=0, p($$P$$|r)=1$$ and *p*(S$$|r)=0$$. For each set of conditions, only 2 of the 3 set of actions describe a unique set of conditional rules, for example DCU|*rps* is just a restatement of RPS|*rps*, and identical moves can be generated using either description. For the remainder of this paper, we choose the following 6 conditional rule sets DCU|*wtl*, RPS|*wtl*, DCU|*rps*, DCU|*r*_A_*p*_A_*s*_A_, D_A_C_A_U_A_, D_A_C_A_U_A_|*r*_A_*p*_A_*s*_A_. We call each pairing of condition and action set a framework. Each framework has $$3 \times 3 = 9$$ set of rules and 6 unique probabilities. We will search for evidence of rule use by humans by testing the $$6 \times 9 = 54$$ candidates from the lag-1 set of rules.

### Detecting repertoire rules

Response probabilities have traditionally been calculated over the entire game without considering possible temporal dynamics. We explicitly consider the temporal dynamics of these probabilities as reflecting the switching of rules, and outline its estimation from data. If a particular lag-1 rule (U|*w* to be concrete) is being used at any point of time during the 300-round game, we expect to see an abundance of consecutive wins (not necessarily occurring in consecutive rounds, as outcomes over consecutive rounds may look something like *wtwlltw*) that are followed by Up transition in the game data. Finding all the wins in the data, define RND(win #*n*) as the round index of the *n*th win. To construct a test to quantify this, we first estimate *p*(U|*w*)(round) using sliding windows of size *B*. We set window size $B=3$ win conditions, and note this will be $>=3$ rounds, and variable at different points of the game. We estimate *p*(U|*w*)(RND(win #1)) by using the 1st, 2nd and 3rd wins, and calculate

1 $$\begin{aligned} {\text{p}}({\text{U}}|w)({\text{RND}}({\text{win}} \#1) = \frac{\#{\text{Up}}}{\#{\text{Up}} + \#{\text{Not Up}}} = \frac{\#{\text{Up}}}{B} \end{aligned}$$

following those wins. Now slide over to the 2nd, 3rd and 4th wins to estimate *p*(U|*w*)(RND(win #2)) using the same procedure. We hypothesize the rule for win persists in the mind even in interim rounds not matching the win condition, so we intrapolate between rounds that were won to obtain *p*(U|*w*) for all rounds. We then count number of rounds where *p*(U|*w*)(round) > threshold of 0.8 over the whole game. To check the significance, we repeat this process using data with order of the 300 rounds shuffled to create a null distribution of data with move content intact but with temporal structure destroyed. If the number of rounds above threshold (rule shuffle score, RSS) is 98th percentile or higher of this null distribution, we deem U|*w* to be in the repertoire. The choice of threshold value of 0.8 and 98th pecentile are arbitrary, though changing the threshold in the range of 0.75–0.9 and the percentile between 96 and 99 results in quantitative but not qualitative differences. Repeating this for all 54 lag-1 rules, we determine the set of rules that are in the repertoire.

### Detecting rule switches

After we find which rules are in-repertoire, we next detect at which rounds switching to a different rule in the repertoire occurred. As an example of what we expect rule changes to look like, Fig. 4A top trace shows rapid swings in *p*(U|*w*)(round) from nearly 0 to 1 or 1 to 0 around rounds 50, 75, 100, 125, 150, 190, 250, 260. We checked our ability to detect rule changes like this in data, by simulating players that 1) switch between rules shown in Fig. 2B and as a comparison, players that played according to 2) the NME. We refer to the data collected in experiment 1) as RS(n)vAI(i), Fig.2A, with n = 1 or 2 specifying which set of rules used, and i = 11, 14 specifying the mean number of rounds between rule switches that are drawn uniformly near the mean) and in experiment 2) as NMEvAI. In RS(n)vAI(i), we know which of the 54 possible rules are in the simulated HP’s repertoire, and we know the exact moment rule sets were switched. In the NMEvAI, we know there is no conditional structure in the data, as well as no rule changes.

### Correlation of behavioral features to subscores of abridged autism quotient

The Abridged Autism Quotient is a 28 item scale, with 4 subfactors Social Skills (7 items), Imagination (8 items), Routine (4 items) and Switch (4 items), and Numbers and Patterns (5 items). Each item is scored on a 4 point Likert scale, and the scores from each item for each subfactor are summed, while the composite score is the sum of all 28 items. Bonferonni corrected correlation coefficients^57^ between each feature and the subscores were calculated, Fig. 7A. The predictive power these features have of subscores were also investigated using nested cross-validated Lasso^58^. We have little to no preconception as to what features might explain each subscore, and as such we sought an impartial and systematic method to find a sparse set of explanatory features for each subscore. Behavioral features and scores from the HPvAI(AQ) population were split by 4-folds. For each fold, the cross-validated Lasso using L1-regularization^59^ was used to determine optimal regularization parameter $\alpha$ and sparse weights for the features using an inner 3-fold train/test split, and this model was used to predict HP subscores using test features. Care was taken to prevent data-leakage between the inner and outer loops. The median *R*^2^ for the prediction of each subfactor was reported across folds for the data, as well as for 100 realizations of shuffled HP IDs. For each HP ID shuffle, *R*^2^ was calculated for all subfactors with the shuffling fixed.

## Results

### Reproduction of existing results

Our contribution builds on recent research that addresses the existence of serial dependence of moves^37,40,42,44,47^, an expected feature of non-NME play. We first reproduce results of existing research to show that under the identical metrics, the behavior of our population is in line with previous experiments. Lag-1 conditional responses calculated over the entire game and averaged over the population often do not reveal a strong serial-dependence, as components are very near 1/3, as reported by^37,42^, and that we confirm, Fig. 3A. However, differences between responses of healthy and schizophrenic patients^37^, and the relative difference in flexibility of components in non-zero sum versions of rRPS^42^ or when opponents are changed^43^, were detectable using population mean. In agreement with^44^, individual responses showed significant heterogeneity in the responses, though agglomerative clustering did not reveal stereotypical patterns of heterogeneity, Fig. 3B, which shows low-dimensional embedding of the 54 components of the conditional responses using t-SNE, with the coloring of the data points corresponding to cluster labels. Compared to a similar analysis of repertoire rules shown in Fig. 4C where clusters are readily visible, clusters are not apparent in conditional responses. Figure 3C shows the response times (duration of each round) following wins, ties and losses, and reproduces the results obtained by Forder et al.^42^ where response after wins is significantly slower than those after ties and losses, suggesting different neural pathways active in decision making. Figure 3D shows the autocorrelation of wins. Brockbank et al.^40^ reported a similar lack of autocorrelation, arguing against exploitation of opponents using transient move patterns and instead arguing for longer-term adaptation to opponents.

**Fig. 5:**
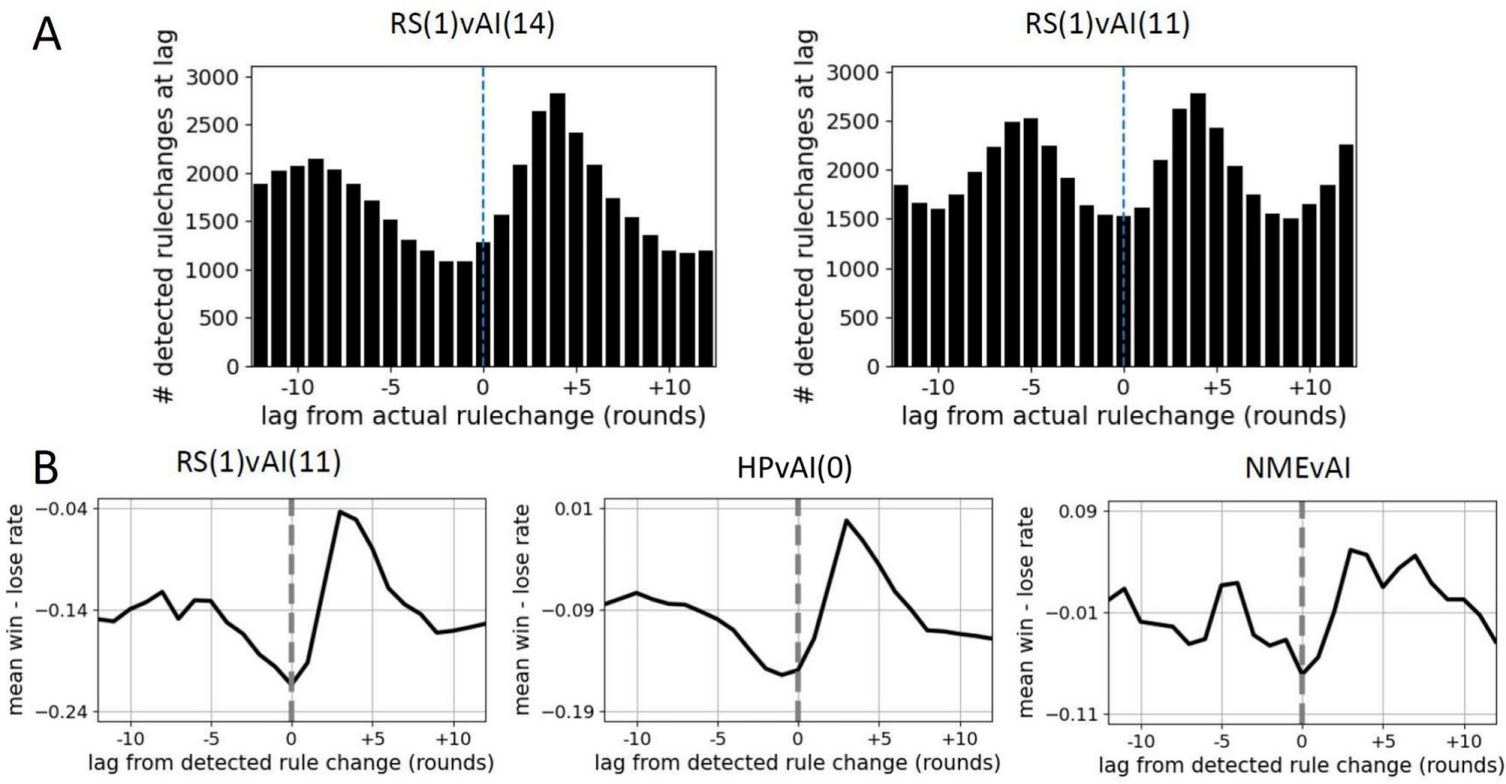
The detection of rule changes and the regaining of advantage. (**A**) Histogram of detected rule change times triggered by ground truth rule change times. The peak is not at lag 0 because rules for each of 3 conditions are changed simultaneously in simulation, but effect of change is not detectable until the conditioning event occurs in data, resulting in an offset of about 4 rounds. Wide peaks indicate the rule change detection method produces many false positives. Nonetheless, the peaks of RS(1)vAI(14) and RS(1)vAI(11) are separated by the respective mean rule change intervals of 14 and 11 rounds. (**B**) Consequences of rule-change. RSTW for HPvAI(0), RS(n)vAI(i), and NMEvAI, were calculated by searching for rule changes in 298, 300, and 300 games, respectively. Prominent increase in wins occurs near instances of detected rule changes in HPvAI(0) and RS(1)vAI(11) (RS(2)vAI(11) similar, not shown) but much less in NMEvAI, as expected. The RSTW for NMEvAI shows some large amplitude near lag 0, but this is due to the much smaller number of events being used in the averaging used to calculate RSTW. Rule changes are detected in NMEvAI, but in a much smaller fraction of games (100%, 94% and 34% of the games, respectively), and even in games where detected, the number of rule change events themselves are significantly smaller as well (80, 38, 3 per game, respectively), and consequently the number of triggering events used in RSTW for NMEvAI is an order of magnitude smaller than HPvAI(0) or RS(1)vAI(11). Adjusting the total number of games to have similar number of events used to calculate RSTW for NMEvAI results in a nearly flat RSTW (results not shown).

### Rule repertoire detection

Figure 4B left, middle and right figure compares the fraction of all the data in RS(n)vAI(11), NMEvAI, HPvAI(0), respectively, in which each of the 54 rules was classified as in-repertoire. There is a clear difference in the number of rules in-repertoire detected in the 3 types of data, with the fewest rules in-repertoire detected in NMEvAI, as expected. The identities of the detected in-repertoire rules are also found to be reasonably close to what is actually in the repertoire for RS(n)vAI(11). Also, the probability of any given rule detected as in-repertoire is much smaller and more uniform in NMEvAI, as expected. HPs, unlike the population of simulated rule-switchers that all had identical rule repertoires, presumably have their own individual repertoires, and hence the fraction of any rule being in-repertoire is much lower in HPvAI(0) population than RS(n)vAI(11) simulated population.

There is clearly structure present in Fig. 4B for HPvAI(0) that is potentially informative about the cognitive process of human competition in rRPS. The C|*w* component is often in-repertoire, while the C|*l* component seldom is. This result is reminiscent to that found by Forder et al. and Srihaput et al.^42,43^ that showed the C|*l* component being flexible in different competitive conditions. The 2nd row shows none of the rules from RPS|*wtl* have a high probability being in-repertoire in HPs, suggesting that HPs likely think of moves in terms of transitions. This is reasonable considering the cyclic dominance of the RPS hands. The 3rd and 4th rows of Fig. 5B show transitions relative to HPs own hands. We see the fraction of transitions D,C,U that are in-repertoire, to be about equal to each other across all trigger conditions. This is reasonable since there should be no preference for any transition direction relative to HP’s own last hand under cyclic dominance. In the 5th and 6th rows, D_A_C_A_U_A_ are transitions with respect to AI’s last hands, and correspond to the least, 2nd and best Cournot responses^37,40^. Interestingly, in every condition, the rank of the frequency of the rule being in-repertoire are the same as its Cournot response rank.

Figure 4B shows that at the population level, a post hoc justification for which of the rules are often found to be in-repertoire can be made from considerations of the rules of RPS. At the same time, it is likely that there are considerable individual variations in what is actually in each HP’s repertoire. Figure 4C shows clustering of pattern of in-repertoire rules by using the percentile of time spent above threshold for each conditional probability timeseries, calculated for each framework as features. This data was projected onto a low-dimensional manifold, where clear spatial clusters were visible. These clusters generally coincided with labels obtained from Agglomerative Clustering of the same features, suggesting that the pattern of in-repertoire rules fits one of a few stereotypical patterns. Figure 4C bottom row for cluster 8 for DCU|*wtl* show a contrarian group of HPs for whom C|*l* is in-repertoire, and cluster 7 shows a group who do not use any C rules in-repertoire. D_A_C_A_U_A_ transitions show groups who switch the best Cournot rules, and other groups who switch the 2nd best Cournot rules.

**Fig. 6:**
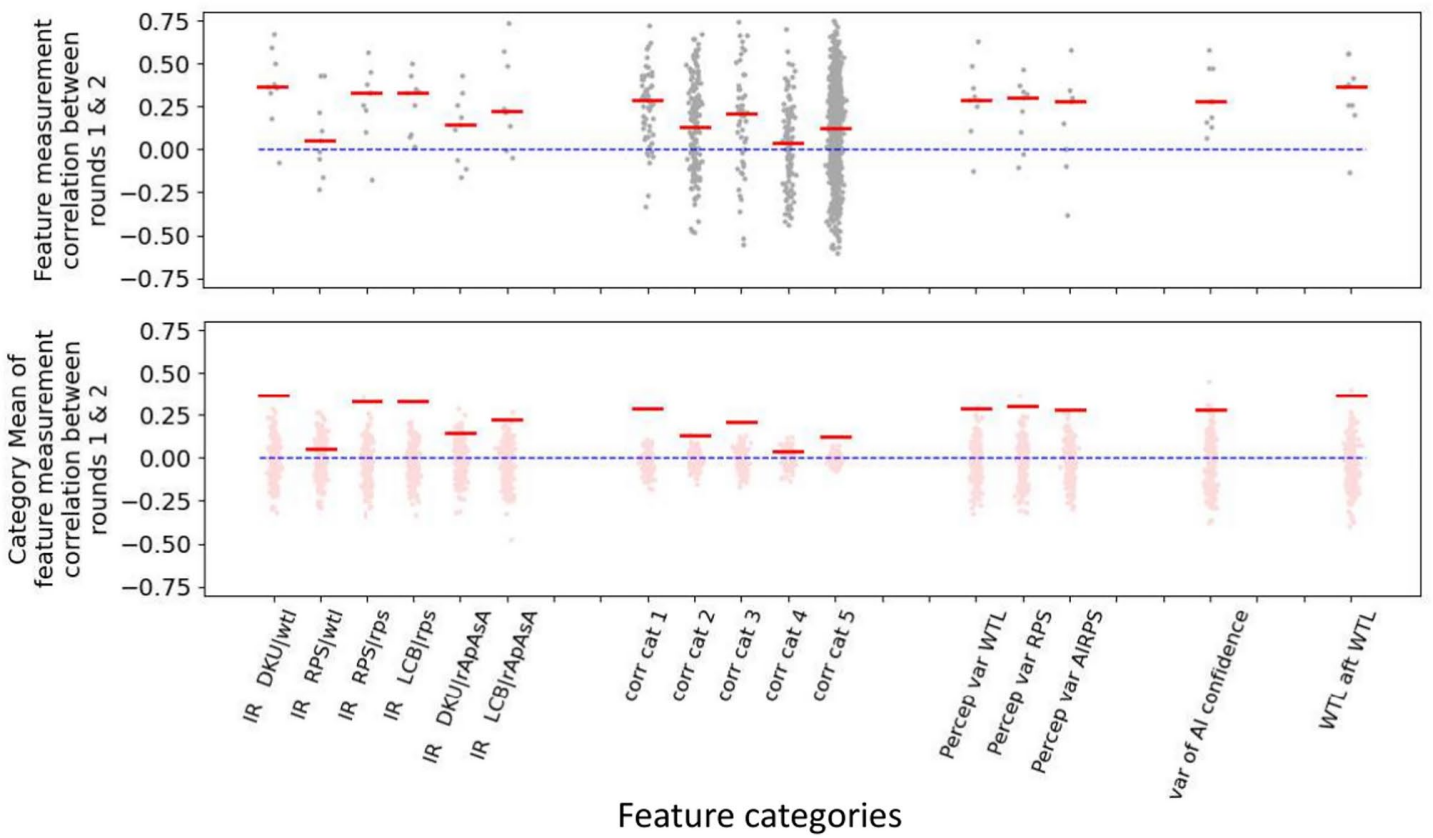
Correlation between the 1st and 2nd measurement of features in HPSvAI(AQ). Features systematically generated and organized into categories measured from 15 subjects who played two 300-round games. Top plot shows the correlation between the 1st and 2nd measurement for features in each category. Red line is the mean correlation for all features in the category. Bottom plot shows the mean correlation for different shuffles of the subject index of the 2nd measurement(pink), while red is the same unshuffled mean copied from top plot. Blue line in both top and bottom is 0.

### Rule change detection

To test our ability to detect rule-switch times from data, we compared the known rule-switch times in the simulated RS(1)vAI(i) data to the detected rule-switch times, Fig. 5. Though we found a large number of false positive detections, Fig. 5, both the rule change times and the mean interval between rule changes were also detected correctly, both important features of the observed rule-switching. The current work seeks to demonstrate the plausibility of the rule-switch hypothesis and narrow down the model class for future work that would improve upon the current detection method.

### Advantage regained at rule change

An expected consequence of episodic rule changing employed against an opponent constantly looking to exploit, is that advantage is temporarily regained immediately after a rule change. Because the AI is constantly updating its internal model of the HP, sudden changes in HP behavior should result in the internal model of the AI becoming obsolete. We define the rule-switch triggered net wins (RSTW), constructed by stacking timeseries segments of outcomes preceding and following detected rule switches. At each lag from the triggering rule switch, the number of losses is subtracted from the number of wins at that lag, and is divided by the combined number of wins and losses,

2 $$\begin{aligned} {\text{RSTW}}({\text{lag}}) = \frac{\#{\text{wins at lag}} - \#{\text{losses at lag}}}{\#{\text{wins at lag}} + \#{\text{losses at lag}}}. \end{aligned}$$

Figure 5A right shows RSTW abruptly increasing at moment of rule change, as expected of a cat and mouse dynamic. Brockbank et al.^60^ looked at the autocorrelation of wins, Fig. 3, butdid not see evidence of streaks of wins and losses, which may have been because of human dyads being used. AI plays consistently throughout the game, likely allowing the consequences of rule-switching to be visualized through the RSTW. We emphasize here that the RSTW is not only a reflection of rule-switching, but also the constant learning of the AI. Pitting the same rule-switcher used in RS(1)vAI(11) against an NME will result in rule switches being detected at the same rate as in RS(1)vAI(11), yet the RSTW will be flat.

### Deriving features from RPSvAI game data

We have presented evidence that the time-dependent conditional response probabilities is a representation of social cognition underlying competitive rRPS, and we have shown that rule-change points can be inferred from them. We hypothesize that subtle behavioral features that reflect the individuality and traits of the HPs can be numerically derived from our data, and that they can potentially be used to differentiate behavior between healthy and afflicted patients. We define candidate features that capture the A) rule repertoire and B) the interplay between the rules in the repertoire. Because the AI is constantly attuned to the most recent moves and the regularities present in them, we looked to C) the internal state of the AI as another source of objective behavioral features. Finally, we included D) conditional outcomes following *wtl* as an easily measurable feature of the possible difference in decision-making pathways of the win and lose condition.

More concretely, A) are the standard deviation of the conditional probability timeseries (6 frameworks x 9 rules = 54 features) and B) are the correlation coefficient between pairs of rule conditional probabilities ($54 \times 53/2$ features). We grouped these correlations into terms from 1) same framework, same condition (ie p(D|*w*) and p(U|*w*)), 2) same framework, different condition, (ie p(U|*w*) and p(U|*t*)), 3) different framework sharing condition class, same condition, (ie p(U|*w*) and p(R|*w*)) 4) different framework sharing condition class, different condition, (ie p(U|*w*) and p(R|*t*)) and 5) different framework that do not share condition class (ie p(U|*w*) and p(R|s_A_$s_{\tiny {\text{ A }}}$). We did not account for whether either rule was in-repertoire or not. However for this analysis, we deemed it sufficient. For C), the AI’s internal state is not easy to interpret, but the relative magnitudes of the 3 outputs of the perceptron can be interpreted as confidence in the decisions. We looked at the decision confidence of the AI perceptrons following $wtl$, $rps$, $r_{\tiny {\text{ A }}}$ $p_{\tiny {\text{ A }}}$ $s_{\tiny {\text{ A }}}$ ($3\times 3 \times 3 = 27$ features), and the conditional outcome ($3 \times 3 = 9$ features) for a total of 1521 features.

### Stability of the measured features

If the candidate features we extracted do reflect the individuality and traits of HPs, we expect them to be stable under repeated measurements. In the HPvAI(AQ) dataset, 15 participants played more than one 300-round game of RPSvAI, and the games were spaced apart on average by about 10 minutes. While this is insufficient for a complete investigation of feature stability, we use these data to find preliminary evidence that behavioral features are not noise and that they have a degree of stability in repeated measurement. For each feature, we calculated the correlation between the 15 values of the measurement from the first 300-round game and the measurement from the second 300-round game, Fig. 6. The features were categorized by how they were derived, and we computed a mean correlation for all the features within that category, Fig. 6A. For each feature, we also calculated correlations between the first 300-round game with the features from the second 300-round game of a randomly chosen subject from with in the 15 subjects. The shuffling was repeated 200 times to obtain a null-distribution of correlations.

While the correlations are not large, Fig. 6, we are unaware of other reports of stability of a behavioral feature derived from game data. Also, our features were systematically constructed, and it is likely that not all such features are stable behavioral features. It may also be possible that 300 rounds is not long enough to accurately capture some features, especially how certain rules are coordinated with others. Most players were able to complete a round in under 7 minutes, and longer rounds or possibly multiple rounds are a possibility. Also, better methodology used to find in-repertoire rules and detect rule changes, is an obvious area where improvements are possible that may lead to better quality in the extracted features.

**Fig. 7:**
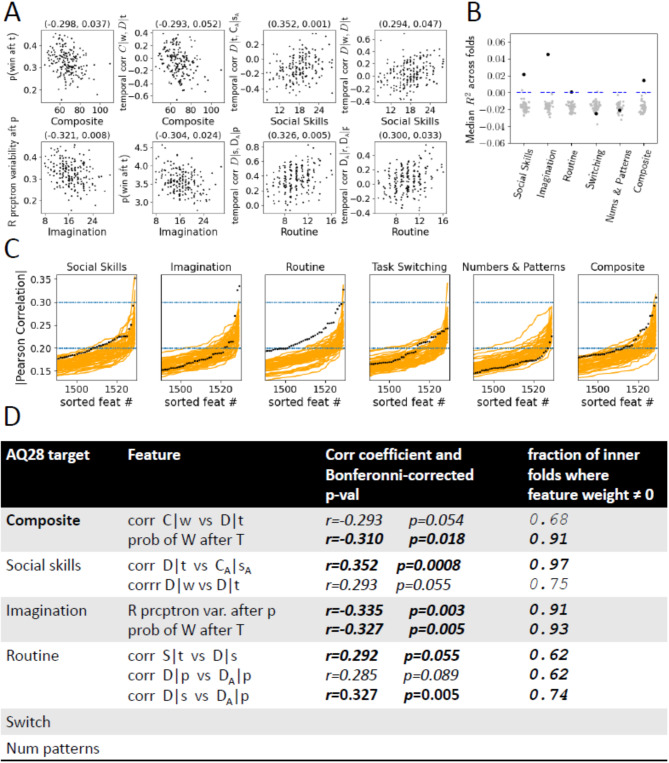
Relationship between features and subscores in HPvAI(AQ) population. (**A**) Scatter plot of features and subfactors whose correlations *r* are significant following Bonferonni-correction. Positive values of the features like “temporal corr | *w*, D|*t*” mean the use of these rules occur concurrently, while negative correlation values mean their use does not overlap in time during the game. (**B**) The sorted magnitudes of all 1521 *r*s (only top 121 shown) between each of the 5 subfactors and 1 composite scores and the features (black). Orange lines show top 121 *r*s between the subfactors and composite score and the features, but where the HP identities of the features are shuffled 50 times. For Social Skills, Routine, Task Switching and Composite, there are a relative abundance of features with large correlations with the respective subscores. (**C**) Mean *R*^2^ scores across folds of predictive models of each subfactor (black), and the mean scores across folds for predictive models with shuffled participant IDs (grey). (**D**) List of features with large correlations with subfactors, the corresponding Bonferonni-corrected *p*-values, and the fraction of folds where Lasso weights were non-zero.

### Game play features reflect player traits

RPSvAI game data features are significantly correlated to and have predictive power for individual AQ28 and its subfactors, the self-reported measure of traits of autistic individuals, Fig. 7A. The magnitude of all 1521 correlations were sorted and compared to the null hypothesis to show that there is a relative abundance of large correlations between the features and Social Skills, Routine and Composite, even if they are not necessarily significant after Bonferronni correction. Fig. 7C shows results of the test for predictive power these features have for the subfactors. The mean coefficient of determination *R*^2^ over all folds of the cross-validated Lasso models for each subfactor shown, with the mean *R*^2^ values shown in blue. The means were $> 0$ for Social Skills, Imagination, Routine and the Composite Score, and significantly different than the the null distribution of mean *R*^2^ for the data where participant ID was shuffled (100 shuffles), shown in grey.

The *R*^2^ can take a maximum value of 1, and also arbitrarily large negative values as well, while $R^2 = 0$ when the mean value of the subscores is correctly predicted. The predictability of the shuffled data are almost always $< 0$, that is shuffled data are not able to predict the mean value of the subscores. Table of features with large magnitude of correlation with subfactors, and the corresponding fraction of folds in which those features had nonzero weight in cross-validated Lasso model is shown in Fig. 7D. The analysis suggests rRPS features are most related to an affinity a subject has to social situations, the ability to read the intentions of others, and whether a subject prefers spontaneity. The rRPS features do not seem to be correlated with a subject’s preference for multitasking over concentration on a single task, or a subject’s attention to numbers and patterns seen in the environment. We also note that in none of the shuffles did we observe a high *R*^2^ in multiple subfactors at once, as seen in the unshuffled data. We had very little intuition about which features might correlate with AQ28 and the subscores, and even with the analysis results, interpreting the results is challenging. However, this is not necessarily a negative aspect of this experiment, as it would be very difficult for a patient to guess what the diagnostic goal of playing rRPS is, let alone understanding and invoking behavior that would support a desired diagnosis.

Our *R*^2^ prediction scores for AQ28 and its subscores appear small in comparison to studies showing 70–80% diagnostic accuracy of ASD^21,27^ using brain signal or video analysis. There are several reasons for this apparent poor performance. First, the goal of this study is not to present a standalone computer-assisted diagnostic solution that differentiates healthy from the afflicted, but rather to demonstrate a spectrum of behaviors in an underexplored domain that has the potential to differentiate patients displaying similar symptoms. Second, our population was recruited anonymously online, and is not a clinical population with matched controls, resulting in poor sampling of the full range AQ28 and its subscores that such a population would likely provide. Third, subjects performed the AIvRPS experiment without any supervision. A fairly uniform time between rounds suggests some participants concentrated during the task, but a significant number of participants exhibited a few long pauses between rounds, suggesting they may have had distractions during the task. Such players also tended to lose more against the AI. Because the HP generally displays dependencies on the previous round, such lengthy pauses are expected to result in poorer quality of behavioral features being extracted as the player is likely to not remember what happened in the previous round after such long pauses. Performing the task in a clinical setting should allow better quality data collection.

## Conclusion

Humans mimic randomness by using the universal mechanism of rule switching in games of rRPS, which is a specific model of how we deviate from the Nash Mixed Equilibrium. There is heterogeneity among individuals about which sets of rules an individual has available and how they are used together. The choice of rules does not follow from rational consideration of the rules of the game, but better reflect what we know as traits. These traits translate into differences in measurable behavioral features of rRPS, and which correlate with the continuum of traits often exhibited by individuals on the ASD spectrum. Together with other genetic, neural and metabolic biomarkers, this social behavioral biomarker may highlight differences between individuals diagnosed as having similar symptoms according to current nosological standards. Because this task can be performed in a clinical setting, it is also compatible with concurrent monitoring of biosignals such as heartbeat and EEG. Further, because this task pinpoints behaviors having limited temporal extent, it may allow identification of time-locked features of neural activity that may help uncover mechanism underlying psychiatric affliction.

For future work, we will build on our discovery of the rule switching mechanism with improvements in analysis technique. We currently detect rules in repertoire and detect switch times with a plausible but ad hoc procedure. We can use this insight to inform a better selection of model class for a latent-state switching statistical model^61^ will be far less prone to noise and false discovery, that will allow us to capture the switching behavior with improved accuracy. We also plan to replace the current rRPS AI with one that better suits the needs of this task. The exploiting AI is crucial in eliciting differentiating behavior, and the 3-perceptron AI has served this purpose, but it is difficult to interpret what the AI is “thinking”. We have found an AI in a kaggle competition (https://www.kaggle.com/competitions/rock-paper-scissors/discussion/221512) that has interpretable states that are similar to our notion of repertoire rules, and may allow us to derive more predictive features from the AI internal state. Replacing our current AI may allow the interpretation of rules chosen by HP in terms of the AI’s internal state, and give clues as to whether rule switches serve to subjective randomness or adversarial reasoning^40^. Being able to elicit behavior that can differentiate healthy and affected states in the laboratory opens up the possibility of observing differences in brain activity. Our work further pinpoints change points in cognitive behavior invisible to the naked eye. Concurrent EEG measurement and employing analysis techniques like global coherence to quantify functional connectivity, is an addition we are currently exploring that may allow us to link neural activity and moment-by-moment changes in cognition^62^ that may reveal cognitive differences of healthy and affected individuals. Finally, we are beginning data collection from a clinical population to observe whether the predictability of AQ scores extends to diagnosed population, and rRPS can differentiate healthy and affected individuals.

## Data Availability

Our data is anonymized and will be made publicly available for use by other researchers at https://github.com/AraiKensuke/AIiRPS.

## References

[1] Kay, S. R., Flszbein, A. & Opler, L. A. The positive and negative syndrome scale (panss) for schizophrenia. *Schizophr. Bull.* **13**, 261–276 (1987). 3616518 10.1093/schbul/13.2.261

[2] Achenbach, T. M. & Ruffle, T. M. The child behavior checklist and related forms for assessing behavioral/emotional problems and competencies. *Pediatr. Rev.* **21**, 265–271. 10.1542/pir.21-8-265 (2000). 10922023 10.1542/pir.21-8-265

[3] Baron-Cohen, S., Wheelwright, S., Skinner, R., Martin, J. & Clubley, E. The autism-spectrum quotient (aq): Evidence from asperger syndrome/high-functioning autism, males and females, scientists and mathematicians. *J. Autism Dev. Disord.* **31**, 5–17 (2001). 11439754 10.1023/a:1005653411471

[4] Hoekstra, R. A. et al. The construction and validation of an abridged version of the autism-spectrum quotient (aq-short). *J. Autism Dev. Disord.* **41**, 589–596. 10.1007/s10803-010-1073-0 (2011). 20697795 10.1007/s10803-010-1073-0 PMC3076581

[5] Williams, M. M., Rogers, R., Sharf, A. J. & Ross, C. A. Faking good: An investigation of social desirability and defensiveness in an inpatient sample with personality disorder traits. *J. Personal. Assess.* **101**, 253–263. 10.1080/00223891.2018.1455691 (2019). 10.1080/00223891.2018.1455691 29717901

[6] Norman, R. M. G., Malla, A. K., Cortese, L. & Diaz, F. A study of the interrelationship between and comparative interrater reliability of the saps, sans and panss. *Schizophr. Res.* **19**, 73–85 (1996). 9147498 10.1016/0920-9964(95)00055-0

[7] Russell, P. S. et al. Diagnostic accuracy, reliability and validity of childhood autism rating scale in India. *World J. Pediatr.* **6**, 141–147. 10.1007/s12519-010-0029-y (2010). 20490769 10.1007/s12519-010-0029-y

[8] Regier, D. A. et al. Dsm-5 field trials in the united states and canada, part ii: Test-retest reliability of selected categorical diagnoses. *Am. J. Psychiatry.* **170**, 59–70 (2013). 10.1176/appi.ajp.2012.12070999 23111466

[9] van de Mortel, T. F. Faking it: Social desirability response bias in self-report research. *Aust. J. Adv. Nurs.* **25**, 40–48 (2008).

[10] Rosenhan, D. L. On being sane in insane places on being sane in insane placest. *Science* **179**, 379–399 (1973). 10.1126/science.179.4070.250 4683124

[11] Wong, E. H., Yocca, F., Smith, M. A. & Lee, C. M. Challenges and opportunities for drug discovery in psychiatric disorders: The drug hunters’ perspective. *Int. J. Neuropsychopharmacol.*10.1017/S1461145710000866 (2010). 20716397 10.1017/S1461145710000866

[12] Ono, Y. et al. Auditory steady-state response at 20 hz and 40 hz in young typically developing children and children with autism spectrum disorder. *Psychiatry Clin. Neurosci.* **74**, 354–361. 10.1111/pcn.12998/full (2020). 32155301 10.1111/pcn.12998

[13] Sugiyama, S. et al. The auditory steady-state response: Electrophysiological index for sensory processing dysfunction in psychiatric disorders. *Front. Psychiatry* **12**, 644541. 10.3389/fpsyt.2021.644541 (2021). 33776820 10.3389/fpsyt.2021.644541 PMC7991095

[14] Miller, L. E. & Saygin, A. P. Individual differences in the perception of biological motion: Links to social cognition and motor imagery. *Cognition* **128**, 140–148. 10.1016/j.cognition.2013.03.013 (2013). 23680791 10.1016/j.cognition.2013.03.013

[15] Koelkebeck, K. et al. Theory of mind in first-episode schizophrenia patients: Correlations with cognition and personality traits. *Schizophr. Res.* **119**, 115–123. 10.1016/j.schres.2009.12.015 (2010). 20060686 10.1016/j.schres.2009.12.015

[16] Gold, R. & Segal, O. The bouba-kiki effect and its relation to the autism quotient (aq) in autistic adolescents. *Res. Dev. Disabil.* **71**, 11–17. 10.1016/j.ridd.2017.09.017 (2017). 28987967 10.1016/j.ridd.2017.09.017

[17] Honma, M. et al. Contraction of distance and duration production in autism spectrum disorder. *Sci. Rep.* **9**, 8806. 10.1038/s41598-019-45250-8 (2019). 31217506 10.1038/s41598-019-45250-8 PMC6584662

[18] Rubido, M. D. V. et al. In search of biomarkers for autism spectrum disorder. *Autism Res.* **11**, 1567–1579. 10.1002/aur.2026 (2018). 30324656 10.1002/aur.2026 PMC6282609

[19] McParland, A., Gallagher, S. & Keenan, M. Investigating gaze behaviour of children diagnosed with autism spectrum disorders in a classroom setting. *J. Autism Dev. Disord.* **51**, 4663–4678. 10.1007/s10803-021-04906-z (2021). 33590429 10.1007/s10803-021-04906-z PMC8531110

[20] Gonçalves, N., Costa, S., Rodrigues, J. & Soarres, F. Detection of stereotyped hand flapping movements in autistic children using the kinect sensor: A case study. In *2014 IEEE International Conference on Autonomous Robot Systems and Competitions (ICARSC)* (IEEE, 2014).

[21] Kojovic, N., Natraj, S., Mohanty, S. P., Maillart, T. & Schaer, M. Using 2d video-based pose estimation for automated prediction of autism spectrum disorders in young children. *Sci. Rep.* **11**, 15069. 10.1038/s41598-021-94378-z (2021). 34301963 10.1038/s41598-021-94378-z PMC8302646

[22] Goldani, A. A., Downs, S. R., Widjaja, F., Lawton, B. & Hendren, R. L. *Biomarkers in autism*10.3389/fpsyt.2014.00100 (2014). 10.3389/fpsyt.2014.00100 PMC4129499 25161627

[23] Shen, L. et al. Biomarkers in autism spectrum disorders: Current progress. *Clin. Chim. Acta*10.1016/j.cca.2019.12.009 (2020). 31857069 10.1016/j.cca.2019.12.009

[24] Yap, I. K. et al. Urinary metabolic phenotyping differentiates children with autism from their unaffected siblings and age-matched controls. *J. Proteome Res.* **9**, 2996–3004. 10.1021/pr901188e (2010). 20337404 10.1021/pr901188e

[25] Heunis, T. M., Aldrich, C. & de Vries, P. J. Recent advances in resting-state electroencephalography biomarkers for autism spectrum disorder-a review of methodological and clinical challenges. *Pediatr. Neurol.* **61**, 28–37. 10.1016/j.pediatrneurol.2016.03.010 (2016). 27255413 10.1016/j.pediatrneurol.2016.03.010

[26] Bruining, H. et al. Measurement of excitation-inhibition ratio in autism spectrum disorder using critical brain dynamics. *Sci. Rep.* **10**, 9195. 10.1038/s41598-020-65500-4 (2020). 32513931 10.1038/s41598-020-65500-4 PMC7280527

[27] Dekhil, O. et al. Identifying brain areas correlated with ados raw scores by studying altered dynamic functional connectivity patterns. *Med. Image Anal.* **68**, 101899. 10.1016/j.media.2020.101899 (2021). 33260109 10.1016/j.media.2020.101899

[28] van Nifterick, A. M. et al. Resting-state oscillations reveal disturbed excitation-inhibition ratio in Alzheimer’s disease patients. *Sci. Rep.* **13**, 7419. 10.1038/s41598-023-33973-8 (2023). 37150756 10.1038/s41598-023-33973-8 PMC10164744

[29] Lehmann, K., Maliske, L., Böckler, A. & Kanske, P. Social impairments in mental disorders: Recent developments in studying the mechanisms of interactive behavior. *Clin. Psychol. Europe* **1**, 1–15. 10.32872/cpe.v1i2.33143 (2019).

[30] Craig, A. B., Grossman, E. & Krichmar, J. L. Investigation of autistic traits through strategic decision-making in games with adaptive agents. *Sci. Rep.* **7**, 5533. 10.1038/s41598-017-05933-6 (2017). 28717229 10.1038/s41598-017-05933-6 PMC5514024

[31] Wang, H. & Kwan, A. C. Competitive and cooperative games for probing the neural basis of social decision-making in animals. *Neurosci. Biobehav. Rev.*10.1016/j.neubiorev.2023.105158 (2023). 37019249 10.1016/j.neubiorev.2023.105158 PMC10175234

[32] Wang, L., Huang, W., Li, Y., Evans, J. & He, S. Multi-ai competing and winning against humans in iterated rock-paper-scissors game. *Sci. Rep.* **10**, 1–8. 10.1038/s41598-020-70544-7 (2020). 32807813 10.1038/s41598-020-70544-7 PMC7431549

[33] Premack, D. & Woodruff, G. Does the chimpanzee have a theory of mind?. *Behav. Brain Sci.* **4**, 515–526 (1978).

[34] Yoshida, W., Dolan, R. J. & Friston, K. J. Game theory of mind. *PLoS Comput. Biol.* **4**, 1–14. 10.1371/journal.pcbi.1000254 (2008). 10.1371/journal.pcbi.1000254 PMC2596313 19112488

[35] Jokar, E. & Mikaili, M. Assessment of human random number generation for biometric verification. *J. Med. Signals Sens.* **2**, 82–87. 10.4103/2228-7477.110403 (2012). 23626943 PMC3632045

[36] Schulz, M. A., Baier, S., Timmermann, B., Bzdok, D. & Witt, K. A cognitive fingerprint in human random number generation. *Sci. Rep.* **11**, 20217. 10.1038/s41598-021-98315-y (2021). 34642344 10.1038/s41598-021-98315-y PMC8511021

[37] Baek, K. et al. Response randomization of one- and two-person rock-paper-scissors games in individuals with schizophrenia. *Psychiatry Res.* **207**, 158–163. 10.1016/j.psychres.2012.09.003 (2013). 23017652 10.1016/j.psychres.2012.09.003

[38] Wang, Z., Xu, B. & Zhou, H. J. Social cycling and conditional responses in the rock-paper-scissors game. *Sci. Rep.* **4**, 5830. 10.1038/srep05830 (2014). 25060115 10.1038/srep05830 PMC5376050

[39] Evidence from experimental economics. Xu, B., jun Zhou, H. & Wang, Z. Cycle frequency in standard rock - paper - scissors games. *Phys. A* **392**, 4997–5005. 10.1016/j.physa.2013.06.039 (2013).

[40] Brockbank, E. & Vul, E. Formalizing opponent modeling with the rock, paper, scissors game. *Games* **12**, 70. 10.3390/g12030070 (2021).

[41] Bonawitz, E., Denison, S., Gopnik, A. & Griffiths, T. L. Win-stay, lose-sample: A simple sequential algorithm for approximating Bayesian inference. *Cogn. Psychol.* **74**, 35–65. 10.1016/j.cogpsych.2014.06.003 (2014). 25086501 10.1016/j.cogpsych.2014.06.003

[42] Forder, L. & Dyson, B. J. Behavioural and neural modulation of win-stay but not lose-shift strategies as a function of outcome value in rock, paper, scissors. *Sci. Rep.* **6**, 33809. 10.1038/srep33809 (2016). 27658703 10.1038/srep33809 PMC5034336

[43] Srihaput, V., Craplewe, K. & Dyson, B. J. Switching competitors reduces win-stay but not lose-shift behaviour: The role of outcome-action association strength on reinforcement learning. *Games* **11**, 1–10. 10.3390/g11030025 (2020).

[44] Zhang, H., Moisan, F. & Gonzalez, C. Rock-paper-scissors play: Beyond the win-stay/lose-change strategy. *Games* **12**, 52. 10.3390/g12030052 (2021).

[45] Lee, D., Mcgreevy, B. P. & Barraclough, D. J. Learning and decision making in monkeys during a rock–paper–scissors game. *Cogn. Brain Res.* **25**, 416–430. 10.1016/j.cogbrainres.2005.07.003 (2005). 10.1016/j.cogbrainres.2005.07.003 16095886

[46] Batzilis, D., Jaffe, S., Levitt, S., List, J. A. & Picel, J. Behavior in strategic settings: Evidence from a million rock-paper-scissors games. *Games* **10**, 1–34. 10.3390/g10020018 (2019).

[47] Brockbank, E. & Vul, E. Repeated rock, paper, scissors play reveals limits in adaptive sequential behavior. *Cogn. Psychol.* **151**, 101654. 10.1016/j.cogpsych.2024.101654 (2024). 38657419 10.1016/j.cogpsych.2024.101654

[48] Stöttinger, E., Filipowicz, A., Danckert, J. & Anderson, B. The effects of prior learned strategies on updating an opponent’s strategy in the rock, paper, scissors game. *Cogn. Sci.* **38**, 1482–1492. 10.1111/cogs.12115 (2014). 24646145 10.1111/cogs.12115

[49] Tsuchiya, E., Oki, J., Yahara, N. & Fujieda, K. Computerized version of the Wisconsin card sorting test in children with high-functioning autistic disorder or attention-deficit/hyperactivity disorder. *Brain Dev.* **27**, 233–236. 10.1016/j.braindev.2004.06.008 (2005). 15737707 10.1016/j.braindev.2004.06.008

[50] Robinson, S., Goddard, L., Dritschel, B., Wisley, M. & Howlin, P. Executive functions in children with autism spectrum disorders. *Brain Cogn.* **71**, 362–368. 10.1016/j.bandc.2009.06.007 (2009). 19628325 10.1016/j.bandc.2009.06.007

[51] West, R. L., Lebiere, C. & Bothell, D. J. *Cognitive Architectures, Game Playing, and Human Evolution* 103–204 (Cambridge University Press, 2005).

[52] Matsuda, K. Creative thinking and random number generation test. *Jpn. Psychol. Res.* **15**, 101–108 (1973).

[53] Sally, D. & Hill, E. The development of interpersonal strategy: Autism, theory-of-mind, cooperation and fairness. *J. Econ. Psychol.* **27**, 73–97. 10.1016/j.joep.2005.06.015 (2006).

[54] Germine, L. et al. Is the web as good as the lab? comparable performance from web and lab in cognitive/perceptual experiments. *Psychon. Bull. Rev.* **19**, 847–857. 10.3758/s13423-012-0296-9 (2012). 22829343 10.3758/s13423-012-0296-9

[55] Shinomoto, S. *Theory of Data Analysis, from Prediction to Simulation (Japanese)* (Iwanami Shoten, 2002).

[56] Rutledge-Taylor, M. F. & West, R. L. Using dshm to model paper, rock, scissors. In *Proceedings of the 33rd Annual Meeting of the Cognitive Science Society*, 2341–2346 (2011).

[57] Curtin, F. & Schulz, P. Multiple correlations and bonferroni’s correction. *Biol. Psychiatry* **44**, 775–777 (1998). 10.1016/s0006-3223(98)00043-2 9798082

[58] Pedregosa, F. et al. Scikit-learn: Machine learning in python. *J. Mach. Learn. Res.* **12**, 2825–2830 (2011).

[59] Tibshirani, R. Regression shrinkage and selection via the lasso. *J. R. Stat. Soc. B* **58**, 267–288 (1996).

[60] Brockbank, E. & Vul, E. Recursive adversarial reasoning in the rock, paper, scissors game. In *Proceedings for the 42nd Annual Meeting of the Cognitive Science Society*, 1015–1021 (2020).

[61] Ghahramani, Z. & Roweis, S. T. Learning nonlinear dynamical systems using an em algorithm. In *Advances in Neural Information Processing Systems* (MIT Press, 1998).

[62] Kerr, M. S. D. et al. Risk-taking bias in human decision-making is encoded via a right—left brain push—pull system. *Proc. Natl. Acad. Sci. USA* **116**, 1404–1413. 10.1073/pnas.1811259115 (2019). 30617071 10.1073/pnas.1811259115 PMC6347682

